# A Review of the Long-term Outcomes of Incontinent Diversion in the Pediatric Neurogenic Bladder

**DOI:** 10.1007/s11934-025-01261-9

**Published:** 2025-03-29

**Authors:** Megan Stout, Mark C Adams, Douglass B Clayton

**Affiliations:** Division of Pediatric Urology, Department of Urology, Monroe Carell Jr. Children’s Hospital at Vanderbilt, Vanderbilt University Medical Center, 2200 Children’s Way, Nashville, TN 37232 USA

## Abstract

**Purpose:**

This review offers an evaluation of the limited literature detailing the outcomes of incontinent urinary diversion in the pediatric neurogenic bladder patient. We will discuss the indications for incontinent urinary diversion, procedural options, surgical techniques, postoperative complications, and overall patient impact.

**Recent Findings:**

Incontinent urinary diversions remain a valuable treatment option for patients with neurogenic bladder especially the highly selected patient with few other options. Irrespective of the diversion chosen, postoperative complications do arise, and long-term follow-up is imperative.

**Summary:**

Ensuring the longevity and effectiveness of the diversion is critical, especially in a pediatric patient cohort with multiple long term risks including obesity– a factor that can directly impact reconstructive function. The dearth of available data highlights the need for more longitudinal studies of pediatric patient cohorts to determine true impact and risk related to incontinent urinary diversion.

## Introduction

Neurogenic bladder or neurogenic lower urinary tract dysfunction (NLUTD) in the pediatric population is an umbrella term that characterizes patients with diagnoses or congenital anomalies that contribute to bladder and external urinary sphincteric dysfunction. The most common causes include neural tube defects. Spinal dysraphism accounts for 50% of all neural tube defects and encompasses myelomeningocele, lipomyelomeningocele, caudal regression syndrome, and tethered cord [1]. The presentation and clinical severity of these conditions exist on a spectrum and warrants vigilant management throughout childhood with eventual transition to adulthood.

Long-term bladder management is usually determined through clinical assessment, radiographic surveillance, urodynamic assessment, and patient expectations. Treatment expectations and objectives for patients and family alike may vary throughout life. The priority of neurogenic bladder management is to protect and preserve kidney function. However, other factors influence the impact of neurogenic bladder on individual quality of life including achieving/improving urinary continence, gaining independence with bladder management, minimizing the need for invasive surgery, and preventing the frequency of symptomatic and/or severe urinary tract infections requiring hospitalization [2]. 

## Goals of Lower Urinary Tract Surgery

Two considerations for reconstructive surgery exist for the pediatric patient with neurogenic bladder. The primary indication is protection of the upper urinary tracts from damage, especially in the setting of a high pressure non-compliant bladder unresponsive to maximal medical management (i.e., anticholinergics, beta-3 agonists, and/or intravesical botulinum toxin). Surgery can also improve bladder and bowel continence for the individual improving “social continence” and independence when less invasive interventions have failed. Reconstructive surgery may consist of procedures that increase bladder capacity or compliance, increase bladder outlet resistance, or provide alternative means for bladder emptying. Surgical decision making involves a complete evaluation of the urinary tract via urodynamic assessment, ultrasound evaluation, the ambulatory and functional abilities of the patient, and patient and family preference [2]. 

Many pediatric patients with NLUTD undergo urinary tract reconstruction, with a majority choosing a procedure that provides an opportunity for continence [3–5]. The most common form of continent reconstruction is an enterocystoplasty (bladder augmentation) using ileum. However, in some instances incontinent urinary diversion may be chosen. The indications for incontinent diversion are variable and include temporizing diversion as a bridge to later reconstructive procedures, non-compliance with clean intermittent catheterization (CIC) due to inability or willingness, severe detrusor overactivity, elevated detrusor pressures with small bladder capacity, and bladder underactivity with poor emptying [6–8]. Incontinent diversion can improve urinary drainage and reduce urinary tract infection. The goal of incontinent diversion, in contrast to continent reconstruction, is to improve a patient’s renal risk profile while eliminating the risks of a continent reconstruction reliant on CIC. Below, we have summarized the available literature investigating incontinent reconstruction including cutaneous vesicostomy (CV), ileal conduit (IC), and ileovesicostomy (IV).

## Incontinent Diversion: Options and Outcomes

### Cutaneous Vesicostomy

Cutaneous vesicostomy (CV) is a diversion option that “vents” the bladder to the skin thereby decreasing intravesical and upper urinary tract pressures and preserving renal function [9]. This procedure is considered for patients with multiple indications including worsening upper urinary tract dilation, recurrent urinary tract infections, high grade vesicoureteral reflux, and a high-pressure bladder despite maximal medical management [10]. CV is more often considered in younger patients as a safe alternative until definitive reconstruction can occur.

Overall, CV is a simple and successful procedure for children [11]. The operative technique avoids need for a bowel segment [11–14]. Typically, a transverse incision is made midway between the pubic bone and umbilicus. A transverse or midline rectus incision exposes the transversalis fascia, and a full bladder aids in identification. The transversalis fascia is incised to expose the bladder. The extraperitoneal bladder dissection is aided by traction sutures or Allis clamps. The urachus and dome of the bladder are brought through the fascial defect, and the urachus and umbilical vessels are ligated. A small portion of the bladder dome is excised, commonly 24 French in size. The detrusor is sutured to the fascia in such a position to ensure the posterior wall of the bladder is taut, thus preventing mucosal prolapse. An everting stoma is created at the skin level with several interrupted sutures and a catheter may be placed in the stoma during early healing.

Though largely seen as a temporizing procedure, Hutcheson et al. reported long-term outcomes of a post pubertal cohort of myelomeningocele patients with CV [12]. Of the 23 patients with permanent CV, 18 of underwent surgery prior to age 11, with a mean age of 2.6 and follow-up interval of 13 years. Hydronephrosis resolved in all patients following diversion. The authors considered developmental delay to be a major factor in 72% of the patients that maintained their CV beyond puberty. CV is also effective for patients with challenging or inconsistent social support. Vallasciani et al. reported CV outcomes in a cohort of long-term institutionalized pediatric patients with neuropathic bladders. The authors found that CV provided a simple, well-tolerated, reversible procedure that protected the upper urinary tracts. Of those that underwent CV creation, 100% of the patient cohort demonstrated improved hydronephrosis, with near total resolution of recurrent urinary tract infections and bladder stone formation [16]. Fischer et al. also found comparable results in the durability of CV. In a cohort of 67 spina bifida patients, the average duration of CV was 14.3 years with a functional CV in 36 patients at last follow up. The authors concluded that many patients could maintain CV into adulthood, with good satisfaction and low complications [17]. 

Disadvantages of CV include prolapse, stenosis, and incontinence (which can be more bothersome as children age). Stenosis can be managed with intermittent catheterization in the short-term, however stenosis and prolapse often require definitive CV revision. An additional disadvantage of CV is the inability to create a stomal bud which makes the use of a stomal appliance impossible. The development of bladder calculi is also possible, and while treatment is straightforward through the CV, it is indicative of underlying urinary stasis that should be corrected [8]. 

### Ileovesicostomy

An alternative method for incontinent urinary diversion includes the creation of an ileovesicostomy (IV). Again, as with any incontinent diversion, appropriate patient and family counseling is required to ensure full understanding of the intervention. IV is a reasonable choice for patients with reduced mental, physical, or social capacity to maintain appropriate CIC regimens, but a desire exists for improved bladder management. IV is ideal for patients with a small capacity bladder and detrusor overactivity [7]. The advantage of the IV over the CV is ability to use a stomal appliance which prevents constant urinary leakage and continuous diaper use. Further advantages of IV include the creation of a low-pressure system, potential avoidance of bladder neck reconstruction, elimination of reliance on CIC, and conversion to a continent diversion if desired [6]. Risks of IV include iatrogenic bowel injury and small bowel obstruction. More commonly, stomal stenosis or prolapse can also occur as with most reconstructive procedures involving stoma creation. Additional risks include stone formation from urinary stasis, recurrent urinary tract infections, and upper tract deterioration.

Surgical technique for IV involves isolation of a 10- to 15- cm segment of small intestine, like that of an ileal conduit creation, which is widely anastomosed in an isoperistaltic manner to the dome of the bladder. A rosebud stoma is formed at the skin level. This creates a low-pressure system, with passive drainage of urine through a large channel from bladder to external drainage appliance. Published outcomes of IV in the pediatric population are limited – with most studies highlighting mixed age or adult patient populations [18–29]. Ching et al. reported the first results of IV in a pediatric patient cohort. When compared to reports from adult patients, the authors found similar complication rates for postoperative UTI (22%), wound-infection (11%), bowel-related complication (44%), stomal related issues (22%), and need for additional surgery (11%) [7. The findings are limited by the single institution, retrospective design making comparison of long-term complications to continent reconstruction difficult. Long-term follow up in IV patients is imperative as increasing patient body mass index (BMI) has been associated with increased stomal complications [23, 27]. Obesity is progressive in many patients with neurogenic bladder, and this must be factored into surgical decision-making. Surgeons must consider the length of the bowel segment needed to traverse the obese abdominal wall, a long stomal bud that can sustain further growth of the abdomen, and the stomal location that avoids the developing or established pannus. Tan et al. noted an association between obesity and additional surgery in 27/50 patients following IV [22]. This was a trend towards significance in total complications, however they noted a relationship between BMI and worsened stomal surgery outcomes. Interestingly, the authors noted BMI may underestimate true body adiposity in this patient population and remains a future area to be studied. Husmann et al. reported results that also caution against the use of IV. The authors described increased episodes of urosepsis requiring hospitalization (82%;14/17 patients) and delayed surgical interventions due to complications from ileovesicostomy (88%; 15/17 patients) compared to other forms of diversion. It should be noted that in this description the IV was combined with simultaneous bladder neck closure, which could have contributed to worse or higher risk outcomes overall [29. Taken together, these data highlight why long term follow up after IV is critical for surgical success and upper urinary tract health.

### Ileal Conduit

The ileal conduit (IC) was initially described in the 1930s and popularized by Bricker in 1950 [30]. IC is considered the gold standard urinary diversion for those with muscle invasive bladder cancer, and has been compared against orthotopic neobladder with continent catheterizable channels [31]. Over time, surgeons have applied IC to the neurogenic bladder population, adults and children alike. Indications for IC mirror those described for CV and IV including inability to reliably perform CIC or when a contraindication to receiving a continent reconstruction exists [29–32]. Later, larger series have evaluated the ileal conduit use in pediatric patients, primarily those with spina bifida diagnosis with up to 15 years of follow-up reported. Its relative ease of construction and early positive results led to its wider application in other clinical situations, including the management of patients with NGB [36]. 

However, the initial popularity surrounding IC use in children with NGB declined during the 1970’s and 1980’s. Investigators reported greater than 50% complication rates, most notably chronic infections and progressive upper tract deterioration [37]. Thus, any discussion of ileal conduit diversion in children with neurogenic bladder should review the historical era and aspects of undiversion procedures. These lengthy and complex surgeries highlighted a remarkable era in pediatric lower urinary tract reconstruction [38]. Wacksman et al. reported an initial undiversion series of 11 pediatric patients. Seven of the patients had undergone major reconstructive surgery prior to follow up, while 4 had advanced renal disease despite intervention [39]. These major reconstructive interventions for undiversion consisted of a wide array of complex procedures including need for psoas hitch, ileal interposition, ureteroureterostomy primary re-anastomosis, transureteroureterostomy, and entire kidney mobilization. Multiple other authors have reported their experience of undiversion in the literature, in which they all describe how they successfully transformed an ileal loop conduit to another type of reconstruction, however noting that these operations were technically difficult even in the most skilled surgical hands [40, 41]. Taken together, the literature indicates that undiversion should be approached judiciously, with caution and with the knowledge that these operations are time-consuming, require meticulous surgical technique, and carry high risk of both early and late complications.

Technique for IC creation has remained unchanged over the years, aside from small technical adjustments. However, IC is the most technical of the three procedures described in this review as IC requires adequate dissection of the ureters for reimplantation into a harvested segment of bowel [36]. The ureters are mobilized with careful preservation of the adventitia and blood supply. Starting 15 cm from the ileocecal valve, a 10- to 15- cm segment of ileum is identified that is of adequate length to reach the anterior abdominal wall for stoma formation. Two vascular arcades of the mesentery should be identified and preserved for adequate bowel segment perfusion. The bowel segment is excised, and bowel continuity is reestablished with an ileoileal anastomosis using a variety of techniques. The ureters are spatulated and implanted into the proximal end of the conduit, ensuring a linear, tension-free watertight anastomosis. In some cases, ureteral stents are placed across the anastomoses into the conduit for adequate healing of the anastomotic sites in the post operative period. The distal end of the conduit is then brought to the anterior abdominal wall and the stoma is matured.

In large series of cystectomy and IC diversion, the 90-day complication rates are 64% (with 13% Clavien-Dindo grade 3–5 complications), with ileus, acute pyelonephritis, bowel obstruction, urine leak, and ureteral obstruction being the most common events to occur [42]. Overall, complications occur in over 60% of patients, with number of complications increasing with duration of follow up, making continued surveillance of these patients essential [43]. 

IC is less common in the pediatric neurogenic population compared to adult cohorts due to various long term complication rates including renal deterioration (16–61%), ureteroileal anastomotic stricture (3–20%), upper tract calculi development (1–11%), pyocystis (1–14%), and stomal stenosis (6–48%) [44]. Cass et al. reported a 22-year experience of complications and renal deterioration rates in 139 children with ileal conduits [44]. Of 224 total complications, 114 required surgical correction. Upper urinary tract deterioration occurred in 16.5% of children followed for 10 or more years. Although renal deterioration is a recognized long-term complication of IC in the adult population as well, the possibility of declining kidney function is especially concerning in the pediatric patient with significant future lifespan. Thus, IC is the least favored diversion option.

## Transitional Considerations

When considering the transition of adolescent and young adult patients from pediatric to adult urologic care, one must consider several factors. Non-compliance with bladder management following lower urinary tract reconstruction carries the potential for severe morbidity or mortality. Fortunately, incontinent urinary diversions may allow for a less regimented bladder management routine, thereby reducing a patient’s overall risk. However, follow up remains critical and should be reinforced to all patients. Follow up evaluations permit close monitoring to ensure adequate urine drainage, early detection of postoperative complications, and subtle signs of upper tract deterioration.

## Conclusions

Incontinent urinary diversions remain a valuable treatment option for patients with neurogenic bladder especially the highly selected patient with few other options [7]. Irrespective of the diversion chosen, postoperative complications do arise, and long-term follow-up is imperative. Ensuring the longevity and effectiveness of the diversion is critical, especially in a pediatric patient cohort with multiple long term risks including obesity– a factor that can directly impact reconstructive function. The dearth of available data highlights the need for more longitudinal studies of pediatric patient cohorts to determine true impact and risk related to incontinent urinary diversion.

## Key References

- Thomas J, Clayton DB, Adams MC. *Campbell-Walsh-Wein Urology 12th Edition: Lower Urinary Tract Reconstruction in Children.* Elsevier; 2021.
  - An updated review of lower urinary tract reconstruction in children, including incontinent urinary diversion, in the classic field text of Campbell-Walsh-Wein Urology.
- Vallasciani S, Al Saeedi A, Khalil IA, Mohamed RB, Muneer E, Abdelmaguid N, Pippi Salle JL. Permanent cutaneous vesicostomy: a pragmatic approach to safely manage lower urinary tract dysfunction in pediatric patients with chronic and life-limiting conditions and neuropathic bladders. Front Pediatr. 2024 Jun 25;12:1409608. doi: 10.3389/fped.2024.1409608. PMID: 38,983,461; PMCID: PMC11231433.
  - A review of pediatric patients with Cerebral Palsy and other neuromuscular diseases admitted to long-term care units for palliative care, demonstrating that in this setting cutaneous vesicostomy is a relatively simple and effective procedure.
  - This represents a pragmatic solution for managing complicated lower urinary tract dysfunction in complex long-term institutionalized pediatric palliative care patients with neurogenic bladders.
- Husmann DA, Viers BR. Neurogenic bladder: management of the severely impaired patient with complete urethral destruction: ileovesicostomy, suprapubic tube drainage or urinary diversion-is one treatment modality better than another? Transl Androl Urol. 2020 Feb;9(1):132–141. doi: 10.21037/tau.2019.09.06. PMID: 32,055,477; PMCID: PMC6995926.
  - In severely impaired patients with a neurogenic bladder and urinary outlet destruction, ileovesicostomy compared to conduit diversion is associated with significantly increased incidence of urosepsis and late surgical complications that required operative intervention.

## Data Availability

No datasets were generated or analysed during the current study.
